# Implementation of a lifestyle and life-skills intervention to prevent weight-gain and cardiometabolic abnormalities in people with first-episode psychosis: the Keeping the Body in Mind program

**DOI:** 10.1192/j.eurpsy.2022.359

**Published:** 2022-09-01

**Authors:** J. Curtis, S. Teasdale, R. Morell, P. Wadhwa, O. Lederman, H. Fibbins, A. Watkins, P. Ward

**Affiliations:** 1 South Eastern Sydney Local Health District, Keeping The Body In Mind Program, Bondi Junction, Australia; 2 UNSW Sydney & Mindgardens Neuroscience Network, School Of Psychiatry, Bondi Junction, Australia; 3 University of Sunshine Coast, Mind And Neuroscience - Thompson Institute, Birtinya, Australia; 4 Ingham Institute for Applied Medical Research, Schizophrenia Research Unit, Liverpool, Australia

**Keywords:** First-episode psychosis, Antipsychotics, weight-gain, Metabolic

## Abstract

**Introduction:**

The development of obesity and metabolic abnormalities that seed future ill-health occur early with antipsychotic treatment. In 2013, the 12-week Keeping the Body in Mind (KBIM) pilot lifestyle intervention was delivered to a small sample of youth experiencing first-episode psychosis (FEP) with <4 weeks of antipsychotic exposure in a cluster-controlled design. The control group experienced significant increases in weight (mean 7.8kg) and waist circumference (mean 7.1cm) compared to non-significant increases (mean 1.8kg) in the KBIM group.

**Objectives:**

To evaluate the effect of KBIM as routine care on anthropometry and metabolic biochemistry in a larger sample of youth with FEP across three mental health services.

**Methods:**

This retrospective chart audit was conducted on youth with FEP, prescribed a therapeutic dose of antipsychotic medication, and who engaged with KBIM between 2015 and 2019. Primary outcomes were weight and waist circumference. Secondary outcomes were blood pressure, blood glucose and blood lipids. Outcomes were collected in a pre-post design. Implementation elements were also obtained from the participant’s medical file.

**Results:**

One-hundred and eighty-two people met inclusion criteria. Follow-up data were available on up to 134 people for individual outcomes. Mean number of sessions attended was 11.1 (SD=7.3). Weight and waist changes were limited to 1.5kg (SD=5.3, t(133)=3.2, p=0.002) and 0.7cm (SD=5.8, t(109)=1.2, p=0.23). Nineteen percent experienced clinically significant weight gain. There were no changes to blood pressure or metabolic biochemistry.

**Conclusions:**

The positive outcomes for weight and waist circumference found in the initial pilot study were maintained with implementation as routine care.

**Disclosure:**

No significant relationships.

